# Mapping and validation of a major QTL for primary root length of soybean seedlings grown in hydroponic conditions

**DOI:** 10.1186/s12864-021-07445-0

**Published:** 2021-02-23

**Authors:** Huatao Chen, Giriraj Kumawat, Yongliang Yan, Baojie Fan, Donghe Xu

**Affiliations:** 1 Japan International Research Center for Agricultural Sciences (JIRCAS), 1-1 Ohwashi, 305-8686 Tsukuba, Ibaraki Japan; 2 Institute of Industrial Crops, Jiangsu Academy of Agricultural Sciences, 210014 Nanjing, P.R. China; 3 ICAR-Indian Institute of Soybean Research, 452001 Indore, Madhya Pradesh India; 4 Institute of Crop Germplasm Resources, Xinjiang Academy of Agricultural Sciences, 830000 Urumqi, Xinjiang P. R. China; 5 Institute of Cereal and Oil Crops, Hebei Academy of Agricultural and Forestry Sciences, 050035 Shijiazhuang, Hebei P. R. China

**Keywords:** Soybean, Primary root length, Quantitative trait loci (QTL), Residual heterozygous lines (RHLs), Near isogenic lines (NILs)

## Abstract

**Background:**

The root system provides nutrient absorption and is closely related to abiotic stress tolerance, but it is difficult to study the roots under field conditions. This study was conducted to identify quantitative trait loci (QTL) associated with primary root length (PRL) during soybean seedling growth in hydroponic conditions. A total of 103 F_7_ recombinant inbred lines (RILs) derived from a cross between K099 (short primary root) and Fendou 16 (long primary root) were used to identify QTL for PRL in soybean. The RIL population was genotyped with 223 simple sequence repeats markers covering 20 chromosomes. Phenotyping for primary root length was performed for 3-weeks plants grown in hydoponic conditions. The identified QTL was validated in near isogenic lines and in a separate RIL population.

**Results:**

QTL analysis using inclusive composite interval mapping method identified a major QTL on Gm16 between SSR markers Sat_165 and Satt621, explaining 30.25 % of the total phenotypic variation. The identified QTL, *qRL16.1*, was further confirmed in a segregating population derived from a residual heterozygous line (RHLs-98). To validate *qRL16.1* in a different genetic background, QTL analysis was performed in another F_6_ RIL population derived from a cross between Union (medium primary root) and Fendou 16, in which a major QTL was detected again in the same genomic region as *qRL16.1*, explaining 14 % of the total phenotypic variation for PRL. In addition, the effect of *qRL16.1* was confirmed using two pair of near-isogenic lines (NILs). PRL was significantly higher in NILs possessing the *qRL16.1* allele from Fendou 16 compared to allele from K099.

**Conclusions:**

The *qRL16.1* is a novel QTL for primary root length in soybean which provides important information on the genetic control of root development. Identification of this major QTL will facilitate positional cloning and DNA marker-assisted selection for root traits in soybean.

## Background

The root system absorbs water and nutrients from soil that are essential for plant growth. The phenomenal formation of the robust and extensive root system is extremely important in crop plants, as it ensures the adaptability to the surrounding environment and the improved resource acquisition in the low input environment [1, 2]. However, roots are the hidden part of plants and have high adaptive plasticity in various environments. Therefore, the characterization of the root system requires considerable efforts in field conditions. As a result, studies on root traits are greatly lagging behind those on other up-ground plant traits, and relatively limited genetic studies are reported for soybean root morphology in field conditions.

Soybean (*Glycine max* L. Merr.) is the most important legume crop in the world, providing most vegetable oils and proteins for human consumption. Due to its biological nitrogen fixation ability, soybean is also important in intercropping and crop rotation. Sun et al. [3] suggested that soybean genotypes with early and fast root growth, long main roots, and more extensive lateral roots have high resistance to adversity stress and improved yield. Studies on various root traits in soybean have reported genetic variation in root elongation, total root length, fibrous roots, surface area, root volume, and root diameter [4–7]. Genetic variability was also reported for root mass in response to various abiotic and biotic stress, such as flooding [8], aluminum toxicity [9, 10], iron deficiency [11–13], manganese toxicity [14], phosphorus deficiency [15, 16], and soybean cyst nematode (*Heterodera glycines*) infection [17, 18]. The large variation observed in root traits suggested that the improvement of soybean by the genetic alteration of root traits is feasible.

Because screening soybean for root traits in breeding populations is tedious and expensive due to the difficulties of measuring root characteristics in field conditions, breeding practices targeting the alteration of root traits are extremely difficult to perform. Quantitative trait loci (QTL) analysis allows the identification of the chromosomal regions that condition phenotypic variation in the morphology of roots and identifies the desirable alleles at these QTLs to be used in marker-assisted selection, which could facilitate phenotyping-independent root modification in soybean.

Attempts for the identification of QTLs associated with various root characteristics in soybean have been carried out, and several root trait QTLs have been reported [19–24]. Root trait QTLs were also mapped in varying phosphorous content [25, 26] and under hypoxia [27]. However, considering the diversity of root traits, the QTLs identified for root traits in soybean are very low compared to other agronomic traits. Particularly for primary root length (PRL), only a few QTLs are known [22, 24]. Thus, extensive studies are needed to identify and characterize QTLs controlling root length traits in soybean.

Deep rooting may help plants sustain longer during drought stress by absorbing water from deeper soil layers. Several studies indicated that deep rooting is positively associated with soybean yield during drought stress and might be the underlying mechanism for drought resistance in tolerant genotypes [28–30]. Uga et al. [31] cloned and characterized *DEEPER ROOTING 1* (*DRO1*), a rice QTL controlling root growth angle, and demonstrated that the alteration of the root system architecture improved drought avoidance in rice. This study encouraged us to improve soybean drought tolerance by altering the root system. The identification of genotypes with rapidly elongating taproot in normal growth conditions may allow the determination of their deep rooting ability, and such genotypes can be used to characterize the underlying genetic mechanism of deep rooting in soybean [32]. Based on a preliminary screening for natural variation in primary root growth of soybean, a Chinese soybean cultivar, Fendou 16, was found to have rapidly elongating and longer primary roots. This study was conducted to identify and validate QTL(s) controlling PRL in soybean during seedling growth.

## Results

### QTL mapping for PRL in the K099 × Fendou 16 RIL population

**Fig. 1:**
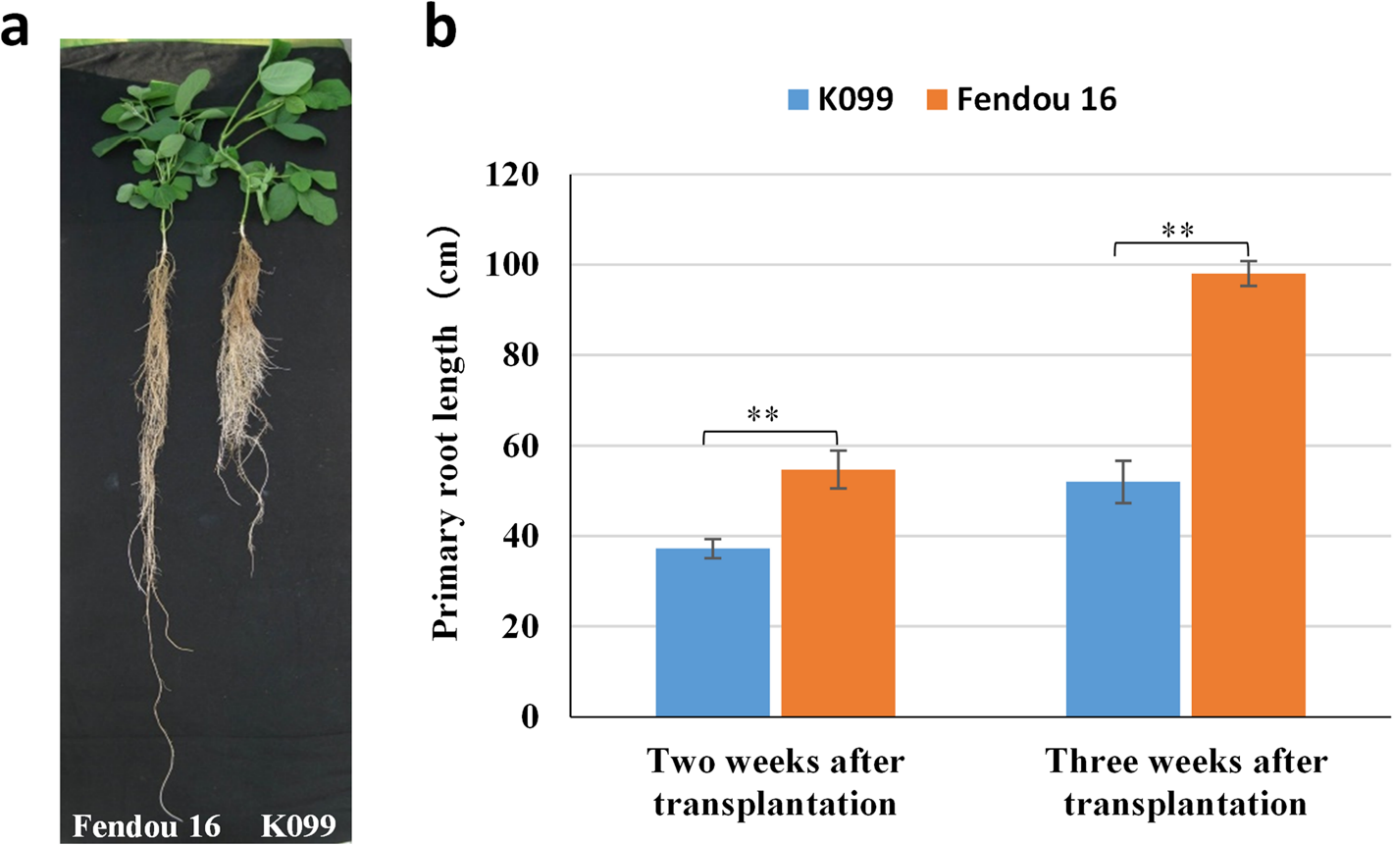
Comparison of root architecture between Fendou 16 and K099 grown in hydroponic conditions after 2 weeks of emergence (**a**), and difference in primary root development between Fendou 16 and K099 at different times after emergence (**b**). Error bars indicate standard deviation (SD; *n* = 8). **P < 0.01 (Student’s *t*-test)

In hydroponic conditions, a significant difference was observed for PRL between Fendou 16 and K099 in the greenhouse experiment at different growth times in the seedling stage (Fig. 1a). The difference in PRL between Fendou 16 and K099 was 17.5 cm at 2 weeks and 46.0 cm at 3 weeks after emergence (Fig. 1b). Although 3-week cultivation could show a big difference in PRL compared to 2-week cultivation, the latter was employed in the mapping population phenotyping experiment, as roots from different genotypes twine together in longer hydroponic cultivation making it difficult to measure the length.

**Fig. 2:**
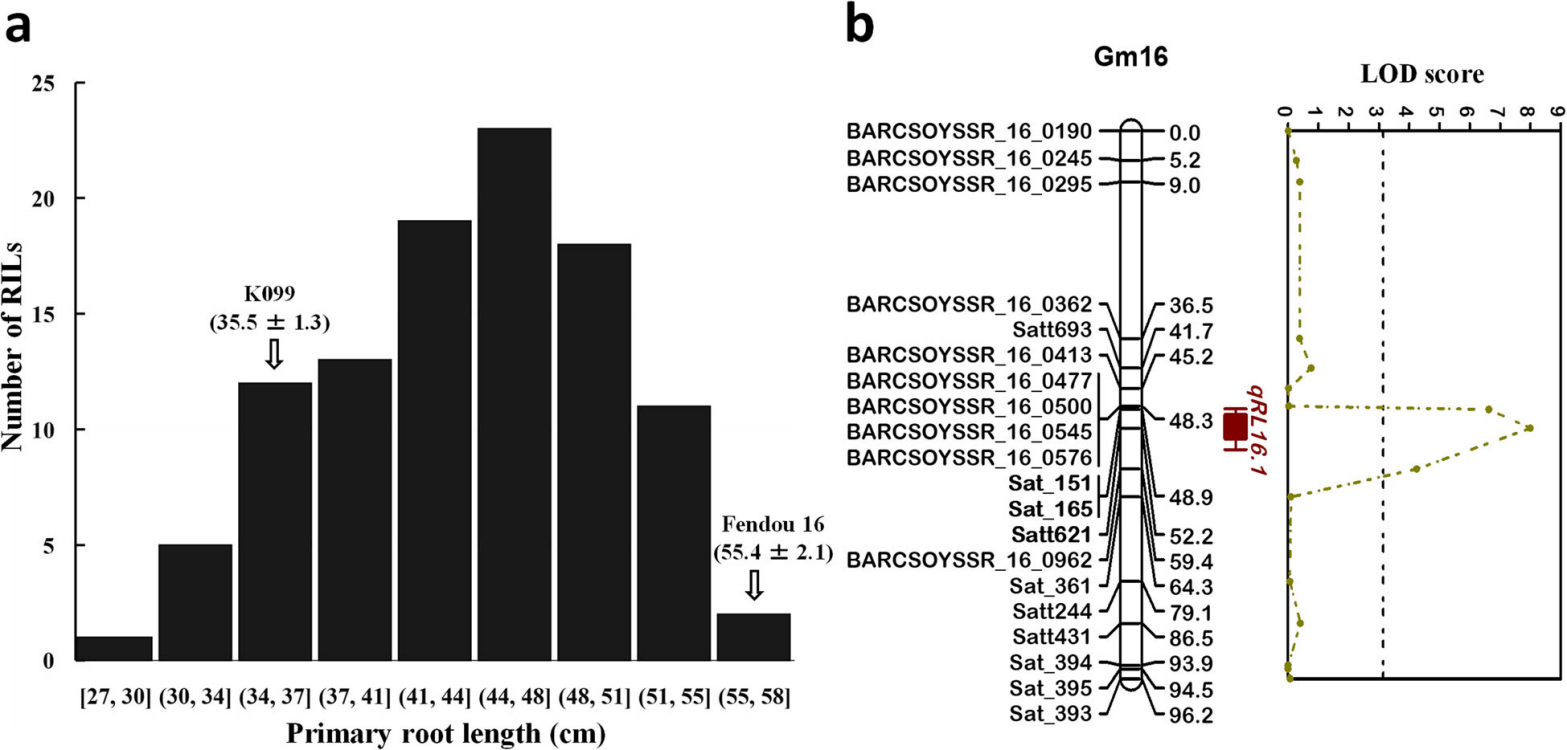
Frequency distribution of PRL in RILs (*n* = 103) derived from K099 × Fendou 16 (**a**), and linkage map of PRL QTL detected on Gm16 in the RIL population derived from K099 × Fendou 16 (**b**). Position of *qRL16.1* is represented by colored bar on the right of chromosome, inner and outer interval of the QTL bar shows 1-LOD and 2-LOD support interval. Dotted black line along X-axis shows LOD threshold as determined by 1000 permutation test (*P* = 0.05)

PRL in recombinant inbred line (RIL) population showed continuous phenotypic distributions and transgressive segregation in both directions was observed (Fig. 2a). QTL analysis by inclusive composite interval mapping (ICIM) method using 223 SSRs genetic map in the RIL population revealed a major QTL for PRL between SSR markers Sat_165 and Satt621 on Gm16 (Table 1; Fig. 2b). This QTL was detected with a high logarithm-of-odds (LOD) score of 7.99 and had a large effect on PRL, explaining 30.25 % of the total phenotypic variation. The additive effect of the Fendou 16 allele increased PRL by 3.4 cm. The QTL was designated as *qRL16.1*. No significant QTL was identified for total root biomass, plant height and shoot biomass traits.

**Table 1:** Chromosome, flanking SSR markers, logarithm-of-odds (LOD) score, coefficient of determination (*R*^*2*^), and additive effects of the QTL identified for primary root length (PRL) in the three mapping populations

Population	QTL	Chromosome	Flanking markers	LOD	*R*^2^ (%)	Additive effect
K099 × Fendou 16	*qRL16.1*	Gm16	Sat_165 – Satt621	7.99	30.25	-3.40
RHLs − 98	*qRL16.1*	Gm16	Sat_165 – Satt621	6.00	24.90	-2.98
Union × Fendou 16	*qRL16.1*	Gm16	BARCSOYSSR_16_0698 – Sat_151	3.25	14.00	-2.23

### **Confirmation of*qRL16.1*** **in a RHLs population**

**Fig. 3:**
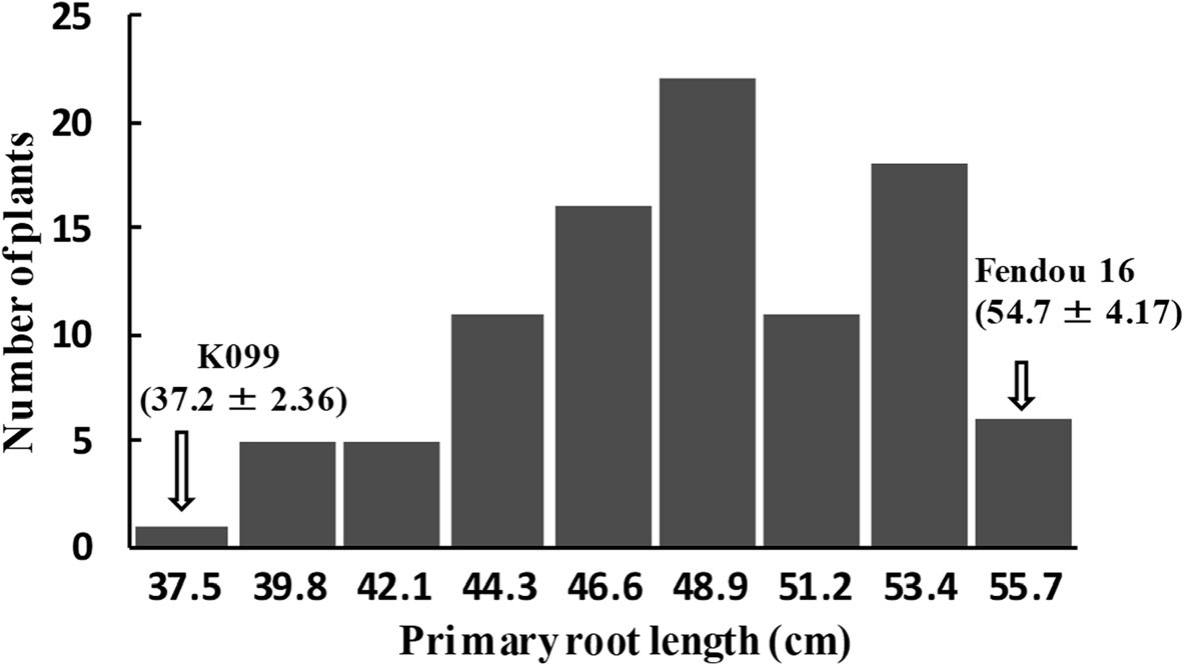
Frequency distribution of PRL in RHLs population (*n* = 97)

A segregating population (*n* = 97) developed by the self-pollination of a residual heterozygous line (RHL), RHLs-98, was used for for QTL analysis. PRL phenotyping and the SSR markers from the *qRL16.1* genomic region were genotyped as per the methods described for the K099 **×** Fendou 16 RIL population. PRL in RHLs population showed continuous distribution (Fig. 3). As a result of QTL analysis, the major QTL for PRL, *qRL16.1*, was again detected between SSR markers Sat_165 and Satt621 on Gm16 (Table 1). *qRL16.1* explained 24.9 % of the total phenotypic variation in this population, and the additive effect of the Fendou 16 allele has increased PRL by 2.98 cm.

### Validation of ***qRL16.1*** in the Union × Fendou 16 RIL population

**Fig. 4:**
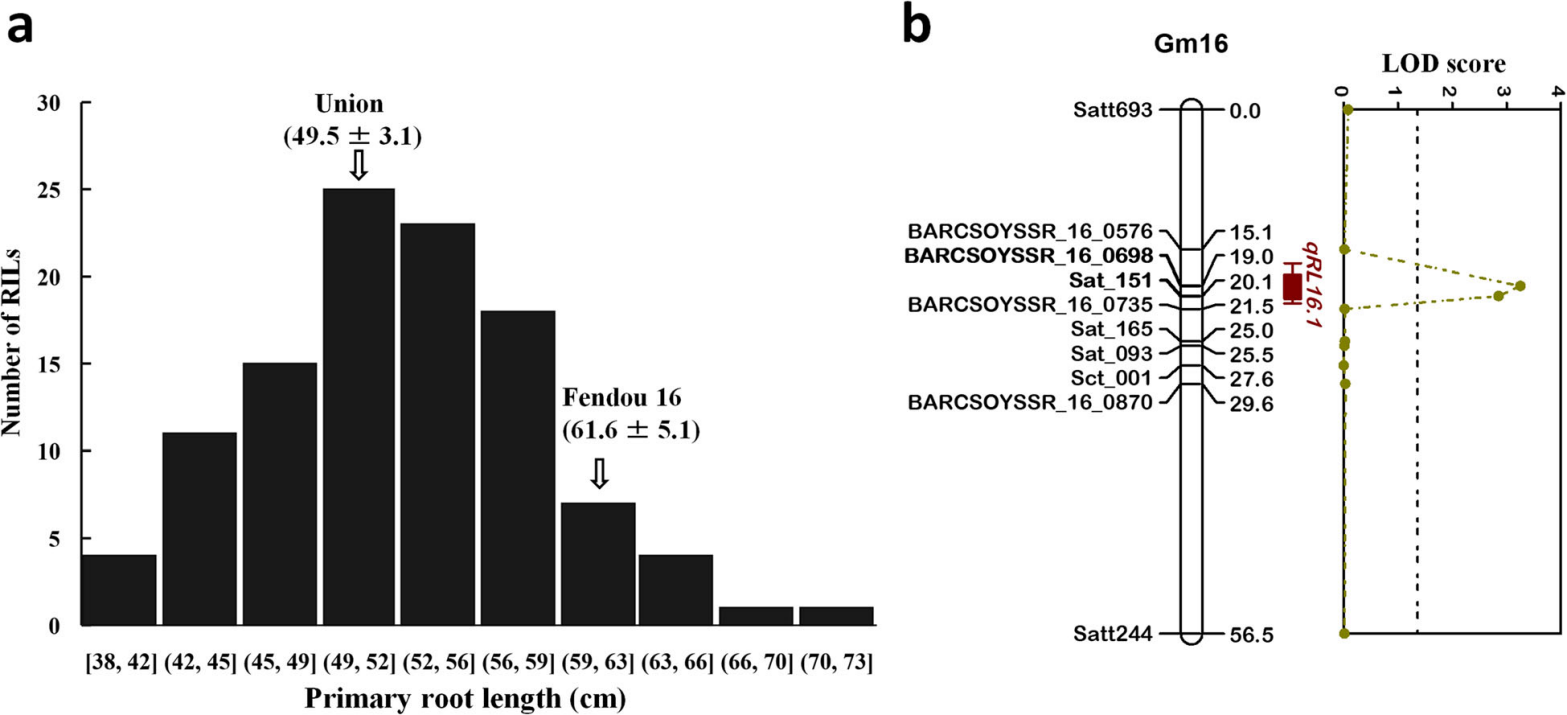
Frequency distribution of PRL in RILs (*n* = 109) derived from Union × Fendou 16 (**a**), and linkage map of PRL QTL detected on Gm16 in the RIL population derived from Union × Fendou 16 (**b**). Position of *qRL16.1* is represented by colored bar on the right of chromosome, inner and outer interval of the QTL bar shows 1-LOD and 2-LOD support interval. Dotted black line along X-axis shows LOD threshold as determined by 1000 permutation test (*P* = 0.05)

To confirm *qRL16.1* in other genetic background, QTL analysis was performed in the F_6_ RIL population (*n* = 109) derived from Union × Fendou 16. RILs along with two parents were evaluated in hydroponic culture, and PRL was measured from soybean seedlings 2 weeks after emergence. Union showed medium-length primary root differing from Fendou 16 by about 12.1 cm. The frequency distribution of PRL in the RIL population is shown in Fig. 4a. Ten polymorphic SSRs from the genomic region of *qRL16.1* were genotyped in this mapping population for QTL analysis. A major QTL explaining 14 % of the total phenotypic variation was detected in the same genomic region of *qRL16.1* (Table 1; Fig. 4b). The additive effect of the Fendou 16 allele increased PRL by 2.23 cm in this population. *qRL16.1* was further confirmed in a different genetic background making it highly useful QTL for marker-assisted breeding of root length in soybean.

### Validation of the effect of ***qRL16.1*** on PRL in its near isogenic lines

**Fig. 5:**
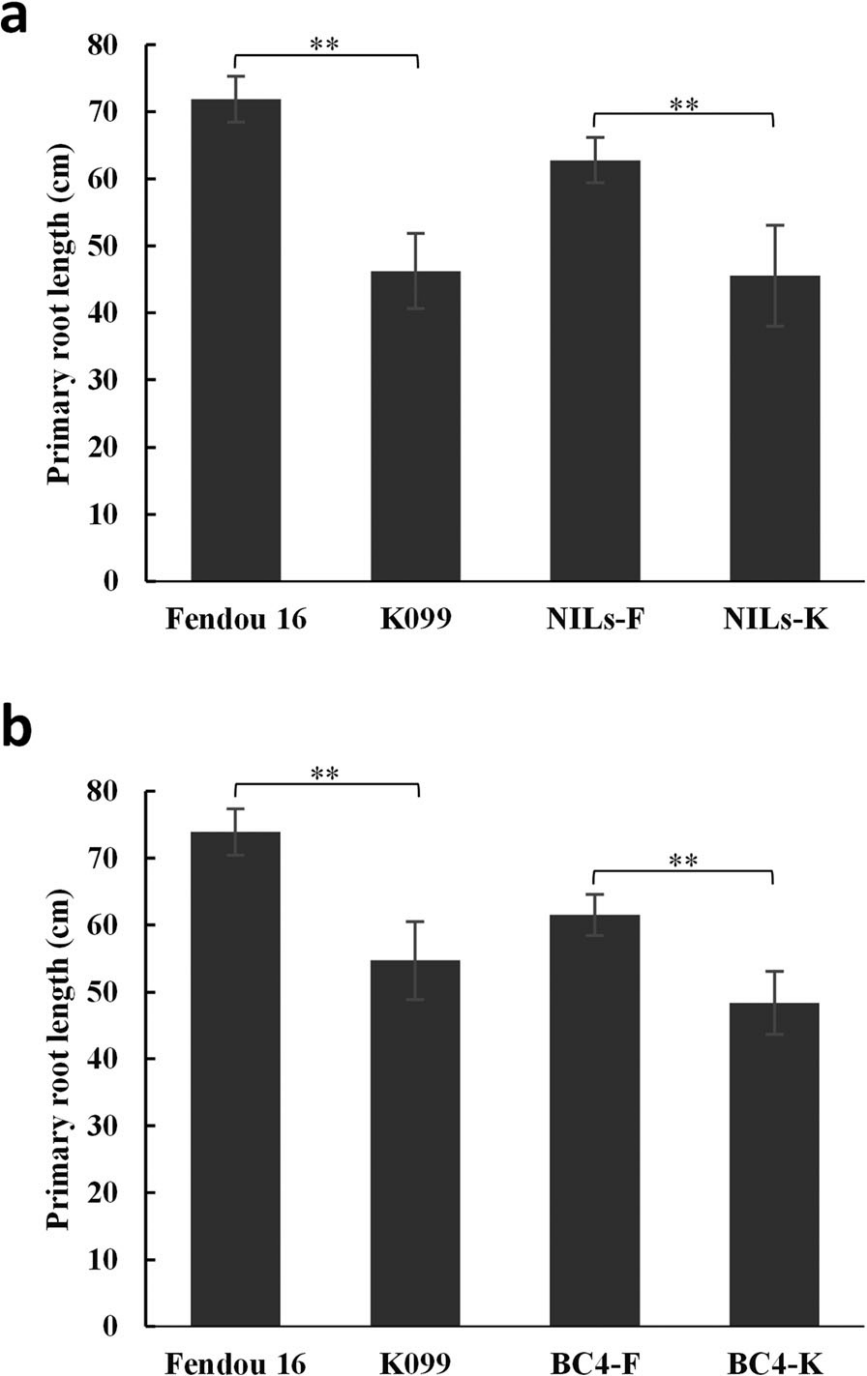
Effect of the *qRL16.1* allele on PRL of two contrasting RHL-NILs, NILs-F and NILs-K, after 2 weeks of emergence (**a**), error bars indicate SD (*n* = 8). Effect of the *qRL16.1* allele on PRL of two contrasting advanced backcross lines, BC4-F and BC4-K, after 2 weeks of emergence (**b**), error bars indicate SD (*n* = 6). **P < 0.01 (Student’s *t*-test)

Two near-isogenic lines (NILs), NILs-F and NILs-K, for *qRL16.1* were developed from the progenies of RHLs-98. These two genotypes had similar genetic backgrounds but differed in the *qRL16.1* QTL region; thus, they may be regarded as NILs. PRL evaluation in hydroponic conditions for RHL-NILs revealed that NILs-F (62.8 ± 3.37 cm) had significantly longer PRL than NILs-K (45.56 ± 7.55 cm), confirming the positive effect of *qRL16.1* on PRL (Fig. 5a). Similarly, two contrasting BC_4_F_3_ backcross lines, BC4-K and BC4-F, differing at *qRL16.1* in the background of K099, were developed by backcrossing and marker-assisted foreground selection for the Fendou 16 allele at *qRL16.1* using SSR markers Sat_165 and Satt621. The evaluation of these two contrasting advanced backcross lines in hydroponic conditions revealed that BC4-F (61.5 ± 3.1 cm) had significantly longer PRL than BC4-K (48.4 ± 4.7 cm), further confirming the effect of *qRL16.1* on primary root development (Fig. 5b).

## Discussion

As the hidden part of plants, roots are difficult to quantify compared to the up-ground traits. Investigation of the root growth performance in hydroponic conditions provides an alternative approach to understand root development. In a hydroponic culture, the whole root system can be obtained for detailed evaluation with minimum efforts. The hydroponic method provides homogeneous growth conditions for the expression of root traits, and QTLs identified in this environment reflect the intrinsic genetic program of root traits in rice [33]. Previous studies using the hydroponic method revealed several root trait QTLs in maize, rice, and soybean [21, 26, 33–37]. A series of studies demonstrated that hydroponics is an efficient method for root morphological investigation and QTL identification.

The growth environmental condition in hydroponics is different from field soil conditions for root development. QTLs or genes expressed in specific environmental conditions, such as drought stress and iron deficiency stress, which occur in field soil conditions, cannot be detected in hydroponic conditions. In this study, a major QTL for PRL in soybean was identified and validated in hydroponic conditions, but of course, the validation of the effect of *qRL16.1* in field soil conditions is necessary for using this QTL in soybean breeding for root trait improvement.

Root trait QTLs have been reported in soybean in previous studies [7, 19–24, 26, 27, 36]. However, only a few studies have reported PRL mapping [22, 24–27]. Manavalan et al. [22] reported that the Satt315-*I* locus on Gm08 contains an essential QTL contributing to early root and shoot growth in soybean, which explained 12.2 % of the phenotypic variation for taproot length in an interspecific backcross-derived inbred line population. Prince et al. [24] mapped two QTLs for taproot length on Gm08 and Gm20. These two QTLs explained 16.2 % of the phenotypic variation for taproot length in an interspecific RIL population. Nguyen et al. [27] detected three QTLs associated with root length in hypoxic conditions on Gm12 to Gm14 in specific environments. Liang et al. [25] identified two QTLs for root length in low phosphorous conditions, explaining 14.4–18.8 % of the phenotypic variation. Cai et al. [26] identified two and seven QTLs for taproot length in high and low phosphorous conditions, respectively. One QTL for taproot length in the low phosphorous condition was identified on Gm16 at 6.6 Mb position. In total, there were only 18 root length QTLs registered in the SoyBase (https://www.soybase.org), but no QTL was reported in the genomic region of *qRL16.1* on Gm16. The QTL detected in this study is a novel QTL conditioning root development in soybean. Further studies on the interaction of *qRL16.1* with other root growth QTLs will reveal the genetic mechanism for root development in soybean.

Fendou 16 and K099 showed a difference in PRL by about 20 cm in the evaluation condition employed in this study. In contrast, PRL between the contrasting pair of NILs and advanced backcross lines for *qRL16.1* was only about 17 and 13 cm, respectively. This *qRL16.1* could not explain the whole variation observed between Fendou 16 and K099. QTL analysis showed that *qRL16.1* only explained 30.25 % of the total variation in the K099 × Fendou 16 RIL population. Therefore, other QTLs/genes might be involved in conditioning PRL in soybean, which are not detected in this study. In the Union **×** Fendou 16 population, *qRL16.1* was detected between markers BARCSOYSSR_16_0698 and Sat_151, explaining 14 % of the phenotypic variation. The lower phenotypic variation explained by *qRL16.1* in this population may be attributed to a lesser difference in the root length of the parents and the involvement of other QTLs. It will be very interesting to identify the causal gene underlying the novel root QTL *qRL16.1*. Low recombination was observed among the markers in the mapped QTL genomic region compared to their physical distance. Despite the small genetic distance between flanking markers (3.1 cM), the genomic region of *qRL16.1* is 2.1 Mb. Such a region is too large for identifying candidate genes. The ongoing fine-mapping of *qRL16.1* might further narrow down the genomic region and enable us to identify a candidate gene for this QTL.

The plant root system is composed of primary root, lateral roots, and root hairs. In this study, only PRL was investigated because, compared to lateral roots and root hairs, PRL is relatively easy to be measured. The primary root growth is less sensitive to nutritional effects than lateral roots and hairs [38]. In this study, no correlation was observed between PRL and total root biomass (data not shown). Moreover, no QTL of total root biomass was detected in the *qRL16.1* region. This result implied that the PRL variation might be due to the fraction of total root length and root volume; that is, longer primary root plants might have few lateral roots, whereas shorter primary root plants might have more lateral roots. Prince et al. [7] identified four loci associated with the lateral root number and distribution of root thickness in diameter class I with a major locus on Gm16. Two single nucleotide polymorphism variations in a gene (*Glyma16.141800*) present near this locus were associated with higher lateral root numbers. The gene reported by Prince et al. [7] is somewhat far from the QTL position detected in this study. The validation of the relationship between *qRL16.1* and *Glyma16.141800* will enable us to understand the mechanism of primary root and lateral root development.

Uga et al. [31] demonstrated that the alteration of the root system architecture improved drought avoidance using *DRO1*, a rice QTL controlling the root growth angle. This study provided a good example of improving drought tolerance through the alteration of the root system. Steele et al. [39] demonstrated that introgression of four root length QTLs into an upland rice cultivar significantly increased yield in a favorable environment. Several studies in soybean also indicated that deep rooting might be the underlying mechanism of drought resistance for tolerant genotypes and is positively related to yield during drought stress [28–30]. Fendou 16, the parental soybean variety used in this study, was originally selected from a landrace genotype which was adopted in a semi-arid area in the middle region of Shanxi Province, China. Based only on data in this study, it cannot be concluded that the long primary root trait in Fendou 16 contributed to its adaptation to drought conditions. Our ongoing study would reveal the effect of PRL on drought tolerance in field conditions.

## Conclusions

A major QTL for PRL in soybean was identified and validated in hydroponic conditions. This study provides an important resource for the alteration of the root system in a soybean breeding program, and for positional cloning of genes controlling root traits. Fendou 16 and the lines developed from it, such as root length NILs, are important materials for studying soybean root development and their interaction with nutrition availability, drought, soil acidity, and other abiotic stress.

## Methods

### Plant materials

A RILs population consisted of 103 F_7_ RILs was used in this study. The RIL population was derived from a cross between soybean cultivars K099 (short primary root) and Fendou 16 (long primary root). Fendou 16 (PI574476A) is a soybean cultivar from Shanxi, China, and K099 is a Korean soybean cultivar. K099 was provided by the National BioResource Project (*Lotus japonicus* and *G. max*; https://www.legumebase.brc.miyazaki-u.ac.jp/). The RIL population was developed from the F_2_ generation by the single-seed descent method without any selection during the generation advance processes.

To confirm the QTL detected in the Fendou 16 × K099 RIL population, a segregating population was developed by self-pollinating a RHL, RHLs-98, which was selected from RILs-98 of the Fendou 16 × K099 RIL population. A total of 97 plants generated by self-pollinating RHLs-98 were used for QTL analysis for PRL.

To confirm the QTL in other genetic background, another F_6_ RIL population (*n* = 109) derived from a cross between Union (PI548622) and Fendou 16 was used for QTL analysis for PRL. Union is an American soybean cultivar with medium PRL.

### Development of NILs

Two NILs, NILs-F and NILs-K, were selected from the progenies of self-pollinated RHLs-98. Homozygous plants with the Fendou 16 and K099 genotypes at the mapped QTL region were respectively selected from the progenies of RHLs-98 based on the genotypes of simple sequence repeat (SSR) markers Sat_165 and Satt621. NILs-F had the Fendou 16 homozygous genotype, and NILs-K had the K099 homozygous genotype at the mapped QTL region. In addition, two advanced backcross lines, BC4-F and BC4-K, possessing contrasting alleles at the mapped PRL QTL region in the background of K099, were developed by backcrossing {((((K099 × Fendou 16) × K099) × K099) × K099) × K099} and foreground selection using markers Sat_165 and Satt621. BC4-F had the Fendou 16 homozygous genotype and BC4-K had the K099 homozygous genotype at the mapped QTL region in the BC_4_F_3_ generation. These two pairs of contrasting NILs were used to confirm the effect of PRL QTL.

### Evaluation of PRL

Hydroponic cultivation was used to evaluate soybean PRL in this study. In brief, soybean seeds for each genotype were sown in a 14 × 14 cm pot filled with vermiculite. About one week's seedlings were transferred into a plastic container filled with 0.5× Hoagland’s culture solution. The seedlings were supported by Styrofoam plates measuring 90 × 60 × 3 cm with 64 holes, each measuring 2.5 cm in diameter and placed 8.5 × 4.5 cm apart. The soybean seedlings were put in the Styrofoam holes and supported by a sponge bar to keep the roots suspended in the solution. Hoagland’s culture solution was constantly circulated by an air pump to supply oxygen to growing soybean plants. Ambient light in the greenhouse was supplemented by high-pressure sodium light for 14 h/day, and the temperature was maintained at 25°C ± 2°C. About 2 weeks after transplantation, all soybean plants were measured for PRL (cm), total root biomass (dry weight basis), plant height (cm), and shoot biomass (dry weight basis). Three plants for each RIL were used for trait measurement in K099 × Fendou16 and Union × Fendou 16 RILs. PRL was measured from cotyledonary node to main root tip and plant height was measured from cotyledonary node to shoot tip, using a ruler. Shoot and root tissues were dried in an oven at 60°C for 72 h, and dry weight was measured in mg. For RHLs population, individual plant of the segregating population was analysed for PRL. For NILs, eight plants of each, NILs-F and NILs-K, and six plants of each, BC4-F and BC4-K, were used for evaluation of PRL.

### DNA marker analysis

Total DNA was extracted from young leaves collected from soybean plants according to the CTAB method [40]. The soybean SSR markers were selected from each linkage group based on the genetic maps of Song et al. [41], Hisano et al. [42], and BARCSOYSSRs [43]. For the K099 × Fendou 16 RIL population, a total of 223 SSR markers, which showed polymorphism between the two parents, were genotyped in the RILs for QTL analysis. For the Union × Fendou 16 RIL population, 10 polymorphic SSR markers were genotyped for the confirmation of the identified QTL.

Polymerase chain reaction (PCR) amplification for SSRs was performed in a final volume of 20 µl with 10 ng template DNA, 10 pmol of each primer, and 10 µl Quick Taq™ HS DyeMix (Toyobo, Tokyo, Japan). PCR was conducted for 35 cycles for 30 s at 94°C, 30 s at 56°C, and 30 s at 72°C and ended after a 5-min extension at 72°C. The PCR products were separated on 8.0 % polyacrylamide gel and stained with ethidium bromide. The band pattern was visualized on a Pharos FX™ Molecular Imager (Bio-Rad, Tokyo, Japan).

### QTL analysis

SSR mapping was performed using the MapDisto version 2.0 software [44]. Loci were assigned to linkage groups based on a logarithm-of-odds (LOD) score of ≥ 3 and a recombination frequency of < 0.45. Map distances (cM) were calculated using the Kosambi’s mapping function. QTL analysis was performed by the inclusive composite interval mapping method using the QTL IciMapping software [45]. The QTL’s significance was estimated from a 1,000 permutations test by random sampling of the phenotypic data. The map positions of QTL on the linkage map was depicted using MapChart software [46].

## Data Availability

The datasets supporting the conclusions of this article are included within the article.

## Abbreviations

ICIM: Inclusive composite interval mapping
LOD: logarithm-of-odds
NILs: Near isogenic lines
PRL: Primary root legnth
QTL: Quantitative trait loci
RHLs: Residual heterozygous lines
RILs: Recombinant inbred lines
SSRs: Simple sequence repeats

## References

[1] Malamy JE, Ryan KS (2001). Environmental regulation of lateral root initiation in Arabidopsis. Plant Physiol.

[2] Lynch JP, Brown KM (2012). New roots for agriculture: exploiting the root phenome. Philos Trans R Soc Lond B Biol Sci.

[3] Sun GY, He Y, Zhang RH, Zhang DP. Studies on growth and activities of soybean root. Soybean Sci. 1996;15:317–21. (in Chinese).

[4] Kaspar TC, Taylor HM, Shibles RC (1984). Taproot elongation rates of soybean cultivars in the glasshouse and their relation to field rooting depth. Crop Sci.

[5] Manavalan LP, Guttikonda SK, Nguyen VT, Shannon JG, Nguyen HT (2010). Evaluation of diverse soybean germplasm for root growth and architecture. Plant Soil.

[6] Fried HG, Narayanan S, Fallen B (2018). Characterization of a soybean (*Glycine max* L Merr) germplasm collection for root traits. PLoS One.

[7] Prince SJ, Valliyodan B, Ye H, Yang M, Tai S, Hu W, Murphy M, Durnell LA, Song L, Joshi T, Liu Y, Van de Velde J, Vandepoele K, Grover Shannon J, Nguyen HT (2019). Understanding genetic control of root system architecture in soybean: Insights into the genetic basis of lateral root number. Plant Cell Environ.

[8] Bacanamwo M, Purcell LC (1999). Soybean root morphological and anatomical traits associated with acclimation to flooding. Crop Sci.

[9] Bianchi-Hall CM, Carter TE, Rufty TW, Arellano C, Boerma HR, Ashley DA (1998). Heritability and resource allocation of aluminum tolerance derived from soybean PI 416937. Crop Sci.

[10] Villagarcia MR, Carter TE, Rufty TW, Niewoehner AS, Jennette MW, Arrellano C (2001). Genotypic rankings for aluminum tolerance of soybean roots grown in hydroponics and sand culture. Crop Sci.

[11] Lin S, Cianzio SR, Shoemaker RC (1997). Mapping genetic loci for iron deficiency chlorosis in soybean. Mol Breed.

[12] Charlson DV, Grant D, Bailey TB, Cianzio SR, Shoemaker RC (2005). Molecular marker Satt481 is associated with iron-deficiency chlorosis resistance in a soybean breeding population. Crop Sci.

[13] Wang J, McLean PE, Lee R, Goos RJ, Helms T (2008). Association mapping of iron deficiency chlorosis loci in soybean (*Glycine max* L Merr) advanced breeding lines. Theor Appl Genet.

[14] Kassem MA, Meksem K, Kang CH, Njiti VN, Kilo V, Wood AJ, Lightfoot DA (2004). Loci underlying resistance to manganese toxicity mapped in a soybean recombinant inbred line population of ‘Essex’ × ‘Forrest’. Plant Soil.

[15] Li JZ, Xie Y, Dai AY, Liu LF, Li ZC (2009). Root and shoot traits responses to phosphorus deficiency and QTL analysis at seedling stage using introgression lines of rice. J Genet Genomics.

[16] Zhang D, Cheng H, Geng LY, Kan GZ, Cui SY, Meng QC, Gai JY, Yu DY (2009). Detection of quantitative trait loci for phosphorus deficiency tolerance at soybean seedling stage. Euphytica.

[17] Miltner ED, Karnok KJ, Hussey RS (1991). Root response of tolerant and intolerant soybean cultivars to soybean cyst nematode. Agron J.

[18] Concibido VC, Denny RL, Boutin SR, Hautea R, Orf JH, Young ND (1994). DNA marker analysis of loci underlying resistance to soybean cyst-nematode (*Heterodera glycines* Ichinohe). Crop Sci.

[19] Abdel-Haleem H, Lee GJ, Boerma RH (2011). Identification of QTL for increased fibrous roots in soybean. Theor Appl Genet.

[20] Brensha W, Kantartzi SK, Meksem K, Grier RL, Barakat A, Lightfoot DA, Kassem MA (2012). Genetic analysis of root and shoot traits in the ‘Essex’ by ‘Forrest’ recombinant inbred line population of soybean. J Pl Genom Sci.

[21] Liang H, Yu Y, Yang H, Xu L, Dong W, Du H, Cui W, Zhang H (2014). Inheritance and QTL mapping of related root traits in soybean at the seedling stage. Theor Appl Genet.

[22] Manavalan LP, Prince SJ, Musket TA, Chaky J, Deshmukh R, Vuong TD, Song L, Cregan PB, Nelson JC, Shannon JG, Specht JE, Nguyen HT (2015). Identification of novel QTL governing root architectural traits in an interspecific soybean population. PLoS One.

[23] Prince SJ, Song L, Qiu D, Maldonado Dos Santos JV, Chai C, Joshi T, Patil G, Valliyodan B, Vuong TD, Murphy M, Krampis K, Tucker DM, Biyashev R, Dorrance AE, Maroof MA, Xu D, Shannon JG, Nguyen HT (2015). Genetic variants in root architecture-related genes in a *Glycine soja* accession, a potential resource to improve cultivated soybean. BMC Genom.

[24] Prince SJ, Vuong TD, Wu X, Bai Y, Lu F, Kumpatla SP, Valliyodan B, Shannon JG, Nguyen HT (2020). Mapping quantitative trait loci for soybean seedling shoot and root architecture traits in an inter-specific genetic population. Front Plant Sci.

[25] Liang Q, Cheng XH, Mei MT, Yan XL, Liao H (2010). QTL analysis of root traits as related to phosphorus efficiency in soybean. Ann Bot.

[26] Cai Z, Cheng Y, Xian P, Ma Q, Wen K, Xia Q, Zhang G, Nian H (2018). Acid phosphatase gene *GmHAD1* linked to low phosphorus tolerance in soybean, through fine mapping. Theor Appl Genet.

[27] Nguyen LV, Takahashi R, Githiri SM, Rodriguez TO, Tsutsumi N, Kajihara S, Sayama T, Ishimoto M, Harada K, Suematsu K, Abiko T, Mochizuki T (2017). Mapping quantitative trait loci for root development under hypoxia conditions in soybean (*Glycine max* L Merr). Theor Appl Genet.

[28] Brown EA, Cavines CE, Brown DA (1985). Response of selected soybean cultivars to soil moisture deficit. Agron J.

[29] Cortes PM, Sinclair TR (1986). Water relations of field-grown soybean under drought. Crop Sci.

[30] Hudak CM, Patterson RP (1996). Vegetative growth analysis of a drought-resistant soybean plant introduction. Crop Sci.

[31] Uga Y, Sugimoto K, Ogawa S, Rane J, Ishitani M, Hara N, Kitomi Y, Inukai Y, Ono K, Kanno N, Inoue H, Takehisa H, Motoyama R, Nagamura Y, Wu J, Matsumoto T, Takai T, Okuno K, Yano M (2013). Control of root system architecture by *DEEPER ROOTING 1* increases rice yield under drought conditions. Nature Genet.

[32] Manavalan LP, Guttikonda SK, Tran LS, Nguyen HT (2009). Physiological and molecular approaches to improve drought resistance in soybean. Plant Cell Physiol.

[33] Wang H, Xu X, Zhan X, Zhai R, Wu W, Shen X, Dai G, Cao L, Cheng S (2013). Identification of *qRL7*, a major quantitative trait locus associated with rice root length in hydroponic conditions. Breed Sci.

[34] Tuberosa R, Sanguineti MC, Landi P, Giuliani MM, Salvi S, Conti S (2002). Identification of QTLs for root characteristics in maize grown in hydroponics and analysis of their overlap with QTLs for grain yield in the field at two water regimes. Plant Mol Biol.

[35] Obara M, Tamura W, Ebitani T, Yano M, Sato T, Yamaya T (2010). Fine-mapping of q*RL6.1*, a major QTL for root length of rice seedlings grown under a wide range of NH^4+^ concentrations in hydroponic conditions. Theor Appl Genet.

[36] Rong Z, Hai-Feng C, Xian-Zhi W, Bao-Duo W, Shui-Lian C, Xiao-juan Z, Xue-Jun W, Zhong-Lu Y, De-Zhen Q, Mu-Lan J, Xin-An Z (2011). Analysis of QTLs for root traits at seedling stage in soybean. Acta Agron Sin.

[37] Kumar B, Abdel-Ghani AH, Pace J, Reyes-Matamoros J, Hochholdinger F, Lübberstedt T (2014). Association analysis of single nucleotide polymorphisms in candidate genes with root traits in maize (*Zea mays* L) seedlings. Plant Sci.

[38] Forde B, Lorenzo H (2001). The nutritional control of root development. Plant Soil.

[39] Steele KA, Price AH, Witcombe JR, Shrestha R, Singh BN, Gibbons JM, Virk DS (2013). QTLs associated with root traits increase yield in upland rice when transferred through marker-assisted selection. Theor Appl Genet.

[40] Doyle JJ, Doyle JL (1990). Isolation of plant DNA from fresh tissue. Focus.

[41] Song QJ, Marek LF, Shoemaker RC, Lark KG, Concibido VC, Delannay X, Specht JE, Cregan PB (2004). A new integrated genetic linkage map of the soybean. Theor Appl Genet.

[42] Hisano H, Sato S, Isobe S, Sasamoto S, Wada T, Matsuno A, Fujishiro T, Yamada M, Nakayama S, Nakamura Y, Watanabe S, Harada K, Tabata S (2007). Characterization of the soybean genome using EST-derived microsatellite markers. DNA Res.

[43] Song Q, Jia G, Zhu Y, Grant D, Nelson RT, Hwang EY, Hyten DL, Cregan PB (2010). Abundance of SSR motifs and development of candidate polymorphic SSR markers (BARCSOYSSR_1.0) in soybean. Crop Sci.

[44] Heffelfinger C, Fragoso CA, Lorieux M (2017). Constructing linkage maps in the genomics era with MapDisto 2.0. Bioinformatics.

[45] Meng L, Li H, Zhang L, Wang J (2015). QTL IciMapping: Integrated software for genetic linkage map construction and quantitative trait locus mapping in biparental populations. Crop J.

[46] Voorrips RE (2002). MapChart: software for the graphical presentation of linkage maps and QTLs. J Hered.

